# Biological and proteomic analysis of a new isolate of the nematophagous fungus *lecanicillium* sp

**DOI:** 10.1186/s12866-023-02855-4

**Published:** 2023-04-20

**Authors:** Lobna Hajji-Hedfi, Wassila Hlaoua, Abdelhak Rhouma, Awatif A. Al-Judaibi, Susana Cobacho Arcos, Lee Robertson, Sergio Ciordia, Najet Horrigue-Raouani, Alfonso Navas, Ahmed M. Abdel-Azeem

**Affiliations:** 1 Regional Centre of Agricultural Research of Sidi Bouzid, CRRA, Gafsa Road Km 6, B.P. 357, Sidi Bouzid, 9100 Tunisia; 2 Department of Plant Protection and Biological Sciences, Higher Agronomic Institute of Chott-Meriem, University of Sousse, Sousse, Tunisia; 3 Department of Biological Sciences-Microbiology Section, Faculty of Science, Jeddah University, Jeddah, 21959 Saudi Arabia; 4 Department of Biodiversity and Evolutionary Biology, Museo Nacional de Ciencias Naturales, CSIC, Madrid, Spain; 5 Dpto Protección Vegetal. Instituto Nacional de Investigaciones Agrarias. Carretera de la Coruña, Km 7, Madrid, 28040 Spain; 6 Unidad de Proteómica Centro Nacional de Biotecnología, CSIC, Campus de Cantoblanco, Madrid, 28049 Spain; 7 Botany and Microbiology Department, Faculty of Science, Suez Canal University, Ismailia, 41522 Egypt

**Keywords:** Choline dehydrogenase, Endochitinase, *Meloidogyne javanica*, Ovicidal, Phylogeny, SCUF Egypt, Tunisia

## Abstract

**Background:**

In our continuing search for biologically active natural enemies from North of Africa with special reference to Tunisian fungi, our teamwork screened fungi from different ecological habitats in Tunisia. Our previous study on the comparative effectiveness of filamentous fungi in the biocontrol of *Meloidogyne javanica*, a taxon (*Lecanicillium*) showed high potentiality against *M. javanica*. We undertook the present study to evaluate the ability and understand the mechanism of this fungal parasite as a biological control candidate against the root-knot nematode *M. javanica*. This study used in vitro bioassays with fungal filtrate cultures, scanning electron microscopy (SEM) observation, and isobaric tag for relative and absolute quantitation (iTRAQ) methodology to characterize the biological and molecular features of this fungus.

**Results:**

The microscopic and SEM observation revealed that *Lecanicillium* sp. exhibited exceptional hyperparasitism against *M. javanica* eggs. The hyphae of this fungi penetrated the eggs, causing destructive damage to the outer eggshell. The exposure to five concentrations of *Lecanicillium* sp. filtrate cultures showed high inhibition of egg hatching, which increases depending on the exposure time; the best results are recorded at 50%, 75%, and 100% dilutions after seven days of exposure. The SEM observation of nematode-parasitized eggs and juveniles suggests that the production of lytic enzymes degrades the egg cuticle and fungal hyphae penetrate unhatched *M.javanica* juveniles. Forty-seven unique proteins were identified from the *Lecanicillium sp.* isolate. These proteins have signalling and stress response functions, bioenergy, metabolism, and protein synthesis and degradation.

**Conclusion:**

Collectively, *Lecanicillium* sp. had ovicidal potentiality proved by SEM and proteomic analysis against root-knot nematode’ eggs. This study recommended applying this biological control candidate as a bio-agent on vegetable crops grown in *situ*.

**Supplementary Information:**

The online version contains supplementary material available at 10.1186/s12866-023-02855-4.

## Background

Root-knot nematodes (RKNs), *Meloidogyne* spp., are harmful polyphagous pests that severely damage crop plants [1]. They are globally distributed, infecting thousands of cultivars and varieties worldwide [2]. *Meloidogyne javanica*, *M. incognita*, and *M. arenaria* are the most common and virulent species in Mediterranean countries, causing significant yield/quality losses in several cultivated crops [3].

Targeted chemical treatments are routinely used to control plant-parasitic nematodes because plant resistance programs have shown unsatisfactory results [4]. However, pesticides are expensive, affect human health, disturb ecosystem equilibrium, and could provoke virulent nematode populations [4]. Because of increased environmental concerns, microorganisms such as fungi are being used as biological controls and an alternative to chemical pesticides [5]. Biological control agents are gaining popularity because they provide safe food and have no adverse effects on the environment [6, 7].

As potential biological control agents, several beneficial fungal species have been investigated; they are considered an environmentally friendly alternative to the chemical nematicides that are currently used on crops [8]. The filamentous fungi of the genus *Lecanicillium* (formerly classified as *Verticillium*) have been proven to have biocontrol capabilities against a range of plant insects and diseases known to affect hundreds of commercially important crops, including aphids [9], whiteflies [10], thrips [11], mealy bugs [12] and powdery mildew [13]. *Lecanicillium* spp. can also be used to control plant-parasitic nematodes [14]. Several species, primarily *V. chlamydosporia* and *V. leptobactrum*, have shown nematicidal activity; *V. lecanii* is one of the most common nematophagous anamorphic Ascomycota on numerous species of nematodes [15, 16].

In our continuing search for biologically active natural enemies from North Africa’ fungi, our teamwork screened fungi from different ecological habitats in Tunisia. Our previous study [14] on the comparative effectiveness of filamentous fungi in the biocontrol of *M. javanica*, a taxon (*Lecanicillium*) showed high potentiality against *M. javanica in vitro* and *in vivo.* The objectives of this study were (1) to understand this antagonistic fungus’s potential mechanisms by scanning electron microscopy (SEM), and (2) to use two-dimensional electrophoresis (2D GE) and iTRAQ approach to analyze total proteins from the *Lecanicillium* sp. mycelium. The current study can provide important experimental information on the proteome for this *Lecanicillium sp.* isolate and give direct experimental evidence to interpret the relationship between the biological control’s potential and secreted proteins.

## Results

### Identification and phylogeny

**Fig. 1:**
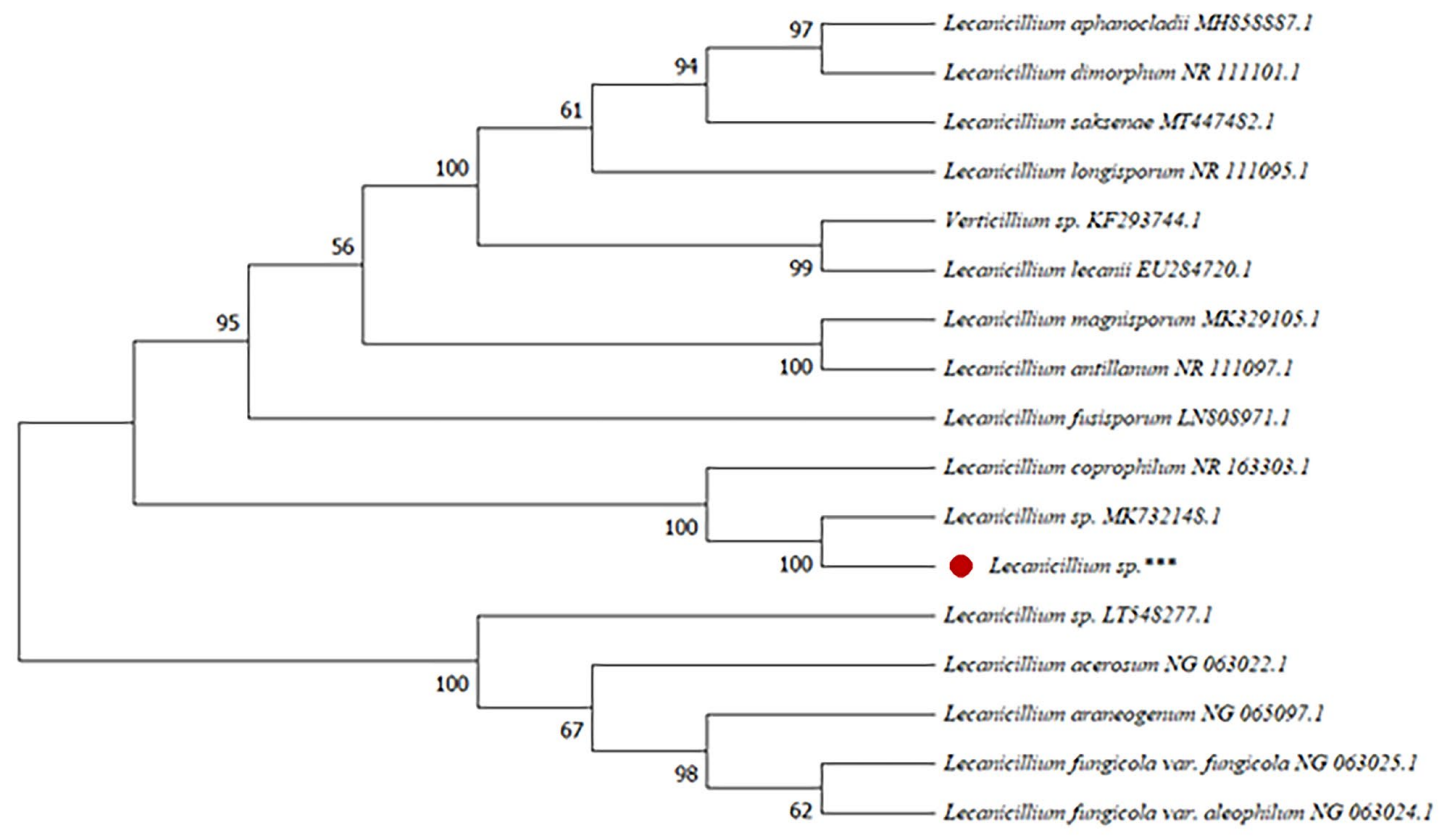
Neighbour-joining phylogenetic tree of 28 S rDNA sequences studied strain (*Lecanicillium* sp.***:OM169327) and its closest phylogenetic relatives. The nucleotide sequences used from representative strains were obtained from the Genbank database under the following accession numbers: *Lecanicillium sp.* (MK732148.1); *Lecanicillium* sp. (LT548277.1*); L. antillanum* (NR_111097.1) *L. fusisporum* (LN808971.1); *L. longisporum* (NR_111095.1); *L. acerosum* (NG_063022.1);*L. araneogenum* (NG_065097.1);*L. coprophilum* (NR_163303.1); *L. dimorphum* (NR_111101.1); *L. fungicola* var. *aleophilum* (NG_063024.1*); L. fungicola* var. *fungicola* (NG_063025.1);*L. lecanii* (EU284720.1); *Verticillium sp.* (KF293744.1); *L. aphanocladii* (MH858887.1); *L. saksenae* (MT447482.1); *L. magnisporum* (MK329105.1). The tree topology was constructed using MEGA11.

The phenotypic identification coupled with molecular identification and blast analysis of sequenced 28 S rDNA gene homology and the phylogenetic analyses based on neighbour-joining (NJ) with 1000 bootstrap sampling revealed that this isolate (OM169327) belonged to the genus *Lecanicillium*. There was a close affinity of 98% to both strains *L. carpophylum* (NR163303) and *Lecanicillium* sp. (MK732148) (Fig. 1).

### Microscopy observations of RKN parasitism

**Fig. 2:**
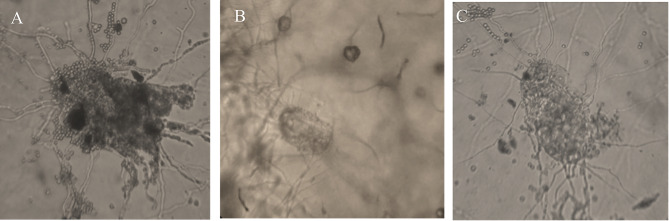
Observation of *M. javanica* infected by nematophagous fungi (**A**: *Pochonia chlamydosporia*; **B** and **C**: *Lecanicillium sp.*; bar = 40 μm) under optical microscopy

**Fig. 3:**
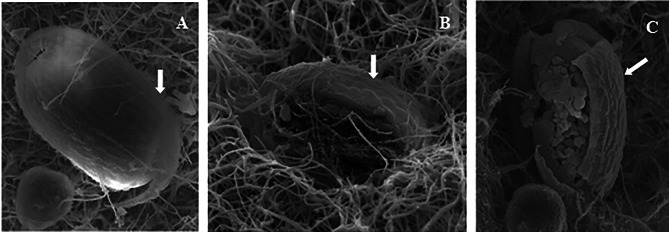
Scanning electron micrographs of *Meloidogyne javanica* eggs treated with *Lecanicillium* sp. (OM169327). **A**, **B** and **C**: scale bars: 50 μm; the white arrow indicates the nematode egg shell

*Meloidogyne javanica* eggs confronted directly with *Lecanicillium* sp. were prepared for light and SEM. Seven days following exposure of *M. javanica* eggs to *Lecanicillium*, microscopic observations showed that antagonistic fungus hyphae adhered to the colonized eggs and inhibited hatching (Figs. 2 and 3 A, and B). The third SEM micrograph showed that the parasitic fungi could break the egg barrier and reproduce inside the eggs by spore production and germination (Figs. 2 and 3 C).

### Effect of culture filtrates on egg-hatching and second-stage juvenile mortality

Although higher concentrations were more effective than lower ones, all tested concentrations were effective in controlling *M. javanica*. Compared to the control, the pure culture filtrate (100%) of *Lecanicillium* sp. was highly effective against root-knot nematode, with a 54% inhibition rate against egg-hatching and a 91% rate of second-stage juvenile (J2) mortality. The 50% and 75% culture filtrate concentrations were almost as effective, with rates of 48.58% and 52.94% against egg-hatching and 91.85% and 90.82% in causing juvenile mortality, respectively (Table 1).

**Table 1:** Effect of culture filtrates of *Lecanicillium* sp. (OM169327) on egg-hatching and larval mortality of *M. javanica*

Fungus Filtrate	Juvenile Mortality	Net Mortality	Egg-hatching Inhibition
**Control**	13.82 ± 4.78 a	–	1.52 ± 1.01 e
**10%**	71.35 ± 7.07 b	66.88 ± 6.97 a	6.76 ± 1.63 d
**25%**	73.04 ± 8.15 b	68.56 ± 9.99 a	29.43 ± 3.52 c
**50%**	91.85 ± 6.43 c	90.68 ± 7.06 b	48.58 ± 3.79 b
**75%**	90.82 ± 4.80 c	89.36 ± 5.49 b	52.94 ± 2.67 a
**100%**	91.55 ± 4.23 c	90.21 ± 4.84 b	53.99 ± 3.27 a

(Means within each column having the same letters are not significantly different; Tukey’s HSD 5%).

### Identification and classification of proteins

Forty-seven proteins were identified, with two peptides at significant levels in the *Lecanicillium* sp. proteome. As contaminant ions, the trypsin- and keratin-derived peptides were excluded from further consideration in MS/MS analysis. Consequently, 22 proteins were unambiguously identified, and their peptide list is provided (Supplementary file). The identified proteins have been characterized from the *Verticillium* genus, including *V*. *alfalfa*, *V. longisporum, and V. dahlia* (Table 2).

**Table 2:** Identified proteins from *Lecanicillium* sp. (OM169327) mycelia

Uniprot_Acc	Description	MW (Da)	pI	Protein score	PSMs	Num. pept	emPAI	Coverage
**EEY14051.1**	actin [*Verticillium alfalfa*e VaMs.102]	41,765	5.45	827	22	11	5.56	44
**CRK20212.1**	hypothetical protein BN1723_012080 [*Verticillium longisporum*]	109,427	7.71	446	11	6	0.26	5.3
**CRK28143.1**	hypothetical protein BN1708_004579 [*Verticillium longisporum*]	270,308	7.92	446	11	6	0.1	2.2
**EEY17447.1**	endochitinase [*Verticillium alfalfae* VaMs.102]	37,004	5.48	110	11	2	0.25	2.9
**EGY17622.1**	alkaline phosphatase H [*Verticillium dahliae* VdLs.17]	72,624	5.31	139	10	2	0.19	3.3
**P00883**	Fructose-bisphosphatealdolase A (MW-Marker) OS = Oryctolaguscuniculus GN = ALDOA PE = 1 SV = 2	39,686	8.31	295	6	5	1.83	42.6
**CRK15627.1**	hypothetical protein BN1708_002817. partial [*Verticillium longisporum*]	39,959	9.21	252	5	4	0.51	10.8
**EGY19081.1**	histone H2B [*Verticillium dahliae* VdLs.17]	14,836	10.12	300	5	4	5.79	32.8
**CRK35690.1**	hypothetical protein BN1708_001322 [*Verticillium longisporum*]	313,673	6.55	225	4	3	0.05	1.7
**EGY19056.1**	nucleoside diphosphate kinase [*Verticillium dahliae* VdLs.17]	16,958	6.84	159	4	3	1.06	20.8
**CRK05543.1**	hypothetical protein BN1708_009694 [*Verticillium longisporum*]	124,836	5.65	127	4	2	0.07	2.8
**CRK10942.1**	hypothetical protein BN1708_009974 [*Verticillium longisporum*]	40,009	5.46	169	3	3	0.51	17.7
**CRK20665.1**	hypothetical protein BN1723_002633 [*Verticillium longisporum*]	361,210	5.71	224	3	3	0.06	2.5
**EGY15746.1**	hsp70-like protein [*Verticillium dahliae* VdLs.17]	71,016	5.09	224	3	3	0.42	11.2
**EEY15502.1**	GTP-binding protein SAS1 [*Verticillium alfalfae* VaMs.102]	25,711	11.33	110	3	2	0.38	14
**EEY16611.1**	choline dehydrogenase [*Verticillium alfalfae* VaMs.102]	68,940	5.12	117	3	2	0.13	3.6
**EEY17079.1**	fumaratereductase/succinatedehydrogenase flavoprotein [*Verticillium alfalfae* VaMs.102]	63,891	6.27	133	3	2	0.21	9.3
**EGY14777.1**	adenosine kinase [*Verticillium dahliae* VdLs.17]	37,916	5.08	131	3	2	0.24	7.5
**EGY15999.1**	GTP-binding protein ypt1 [*Verticillium dahliae* VdLs.17]	22,632	5.31	110	3	2	2.55	46.5
**CRK11434.1**	hypothetical protein BN1708_010158 [*Verticillium longisporum*]	83,965	5.93	95	2	2	0.1	3.7
**CRK35694.1**	hypothetical protein BN1708_001326 [*Verticillium longisporum*]	373,304	8.45	151	2	2	0.02	1
**EEY17295.1**	ATP synthase subunit beta [*Verticillium alfalfae* VaMs.102]	55,705	7.04	122	2	2	0.35	9.3

**Fig. 4:**
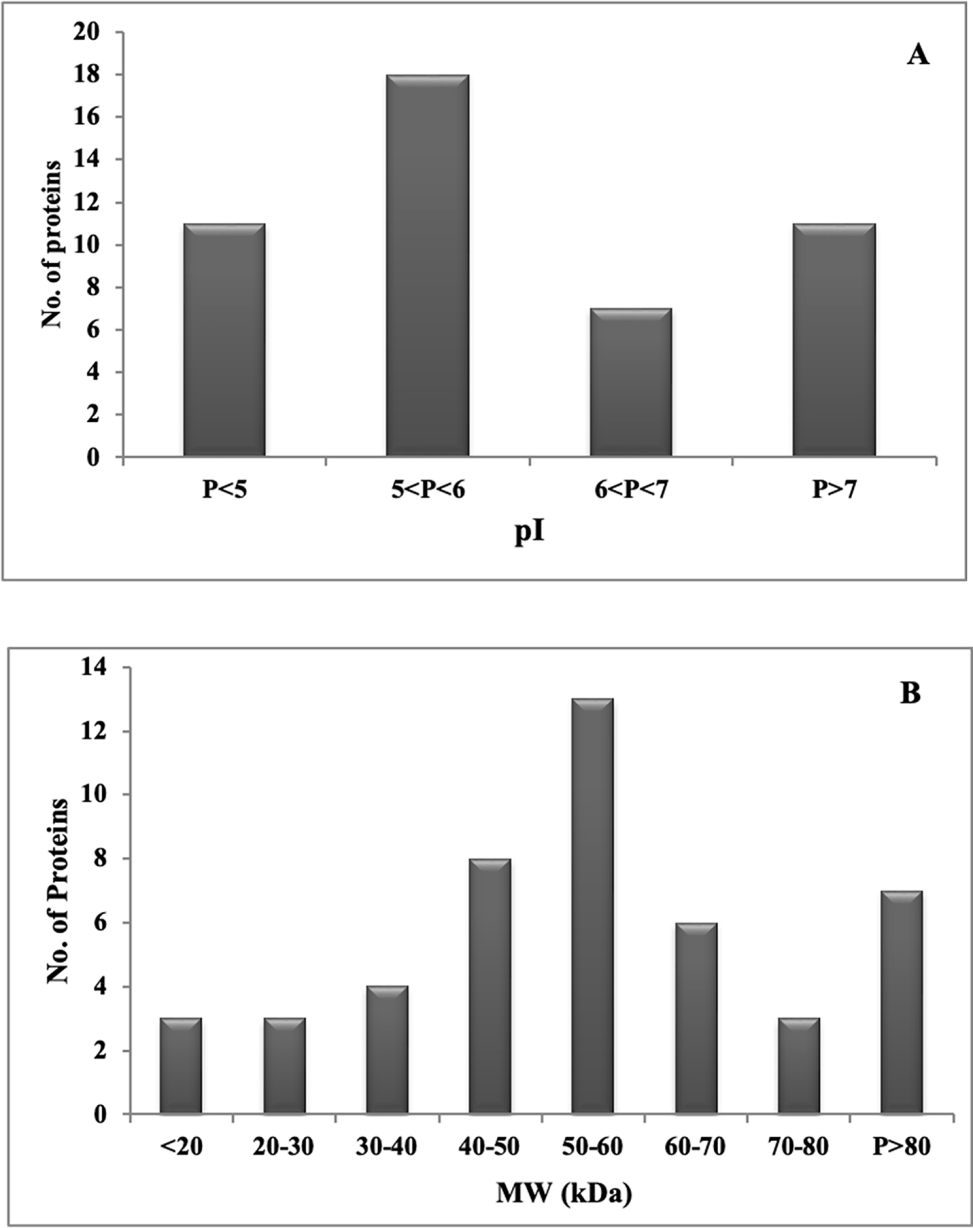
Characteristic features of mycelium proteins of *Lecanicillum* sp. (OM169327). Distribution of the identified proteins in relation to their pI (**A**) and molecular mass (**B**) are shown. The proteins were sorted based on their experimental molecular weight

The identified proteins were then classified based on physiochemical characteristics such as mass and pI. The smallest and the largest molecular masses obtained were 14.83 and 373.3 kDa, respectively. Among the identified proteins, 14 were distributed among the 15–80 kDa molecular mass intervals. Moreover, we found one protein with a mass of fewer than 15 kDa and seven with a mass greater than 100 kDa. The pI of the proteins identified in *Lecanicillium* sp. proteome ranged from 5.08 to 11.33, which is a classic feature of the *Lecanicillium* genus proteins (Fig. 4A and B).

The identified mycelial proteins were functionally classified into five groups: metabolism, energy production, signalling and stress response, protein synthesis and degradation, and other unknown protein functions. Four protein species were identified as being involved in stress response function and signalling: actin, hsp70-like protein, fumarate reductase/succinate dehydrogenase, and GTP-binding protein ypt1. The actin and hsp70-like proteins, in particular, were related to biotic stress and involved in activating specific signalling responses following pathogen perception.

A subset of the identified proteins shared homology with fungal proteins that have roles in energy production and metabolism; the fungus may use these proteins in degradation and nutrient uptake. These classes include proteins such as adenosine kinase, ATP synthase subunit beta, nucleose-binding protein, GTP-binding proteins SASA, fructose-biphosphate aldolase A, alkaline phosphatase H (maybe involved in fungal adhesion and invasion during parasitism), nucleoside diphosphate kinase and histone H2B proteins, which could be involved in the new replication of DNA into the chromatin.

We identified two proteins involved in protein synthesis, assembly, fate and degradation. The primary proteins included in this class were endochitinase, considered a cell-wall degrading enzyme (CWDE), and choline dehydrogenase, another peptidase. Finally, nine proteins were annotated in the databases as hypothetical or putative uncharacterized proteins and one as a conserved domain.

## Discussion

In the ongoing search for biologically viable natural enemies of Tunisian fungi against *M. javanica*, as the dominant RKN worldwide, the current study discovered that *Lecanicillium* sp. is effective in reducing egg hatching and infecting nematode eggs as a safe eco-friendly alternative [17].

Various studies carried out by several investigators like Goettel et al. [18] and Al-Ani et al. [19] revealed that *Lecanicillium* spp. has higher levels of egg parasitism than many other fungal species. The ovicidal potentiality may be referred to that conidia and hypha of nematophagous fungal taxa can easily penetrate the eggs and kill the juveniles [19–21].

Examination of living fungal spores and *M. javanica* eggs under SEM illustrated the host-parasite interaction between *Lecanicillium* sp. and nematode eggs. The first interaction step is the adhesion phase when the fungus adheres to the nematode egg and produces hyphal tips and adhesive conidia that immobilize the host. Later on, the penetration phase, when the production of specific enzymes facilitates the penetration of the egg by the parasitic fungus. According to the bioassays (egg-hatching inhibition and larval mortality) and SEM observations, *Lecanicillium* sp. is effective as an egg parasite, possibly breaking the eggshell using CWDEs, and revealed as an endoparasite as mentioned by several similar studies [22, 23].

In addition to the previous results, culture filtrates of *Lecanicillium* sp. inhibit egg hatching and increase J2 mortality of *M. javanica*. This result supports the findings of Hussain et al. [24], who noted that this nematophagous fungus reduced the level of hatching and increased the mortality of the J2. *Lecanicillium muscarium* successfully decreased juvenile penetration, as demonstrated by Hussain et al. [25], leading to a decrease in gall size and egg masses. Smaller galls and fewer egg masses provided more proof that the nematode was affected directly by nematophagous fungus [26]. Several researchers have discovered that the nematophagous potentiality of different fungal taxa filtrates e.g. *Verticillium, Trichoderma* spp. and *Purpureocillium lilacinus* could be due to the production of toxins, nematicidal metabolites, and lytic enzymes [14, 27–33] respectively.

*Lecanicillium* sp. (OM169327) profile-mapping indicates several hydrolytic enzymes, including choline dehydrogenase and endochitinase, the latter being one of the primary CWDEs. Those enzymes facilitate the biocidal activity of several biological control agents, and they are critical parasitic components as stated by numerous researchers [34, 35]. The CWDEs, including the xylanases, glucanases, lipases, pectinases, chitinases, and cellulases, contribute to fungus virulence towards pathogens and encourage plant root colonization [14, 36].

The potential candidate, *Lecanicillium* sp., proteome contains proteins related to signalling and stress responses that could stimulate plant-host resistance to the pathogen. The biocontrol agent could mitigate the pathogen effect indirectly by boosting the plant defense response through signalling and stress-related proteins [14, 37]. In addition, bioenergy and metabolism proteins were probably involved in space and nutrition competition mechanisms occurring between the antagonistic fungus and the pathogen. The competition action mode was previously described as a biological control mechanism [38].

Proteomic analysis of the potential candidate, *Lecanicillium* sp. recorded some unknown function proteins. This study is directed toward the international attitude to the importance of investigation of the Hyphomycetes proteome as mentioned by Doyle (2011) [39] with special reference to “unknown function proteins”. *Lecanicillium* sp. proteins with unknown functions will be subjected later for further characterization (by means of targeted proteomics as conceptual prove) [40, 41] in order to understand the molecular mechanisms of their biocontrol potential as recommended for similar biocontrol taxa *viz. Verticillium* spp. [39]. In addition, the fungal proteomics allied to the transcriptomic could contribute to understanding more about the proteins’ functions and provide online resources for the functional categorizing of fungal genes and proteins [42].

This study suggests that direct parasitism, production of hydrolytic enzymes, competition, and induction of host resistance are the mechanisms by which *Lecanicillium* spp. inhibit parasites. The numerous mechanisms highlight the remarkable potential of *Lecanicillium* sp. as a powerful biocontrol agent. Several studies have investigated the *Lecanicillium* sp. interaction with the host and parasite and underlined the potential mechanisms that could be involved. They reported that the fungus could penetrate eggshells via hyphae or enzymatic action. This is because nematodes’ eggs contain protein and chitin. Hydrolytic enzymes are responsible for the penetration of the J2 cuticle and the eggs [43–45].

## Conclusions

The complementary use of SEM and proteomics confirmed the biocidal effect of *Lecanicillium* sp. against the plant parasitic nematode *Meloidogyne javanica*. This study described the properties of the proteins within the nematophagous fungus proteome and the proteins’ possible roles. This knowledge will allow the selection of virulent strains of fungi to be used in biocontrol programmes. Further studies are required to show predicted proteins’ predicted changes during pathogenicity and identify determinants playing a role in biological control. This study shows the tested fungus to be a promising biological control candidate that could be involved in integrated pest management strategies. Therefore, we recommend applying this bio-agent to vegetable crops in situ.

## Material & methods

### Nematode preparation

Monoxenic populations of *M. javanica* were maintained on susceptible tomato *cv*. Riogrande in glasshouses at the Higher Agronomic Institute of Chott-Meriem, Tunisia. The eggs were extracted from two-month-old galled roots with hypochlorite sodium (0.5%) for 3 min, as described by Hussey and Barker [46] and collected on an egg-suspension/sugar-flotation gradient (30% w/v). To obtain the *M. javanica* juveniles, nematode egg masses were placed on 1 mm pore-size sieves, lined with a double layer of tissue paper and placed in water-filled 10-cm-diameter Petri dishes. The Petri dishes were incubated for three days, and the water containing the hatched J2s was collected. The numbers of J2s were determined using a stereoscopic microscope, and the eggs and J2 suspensions were used in the experiment’s bioassays.

### Fungus isolation and identification

*Lecanicillium* sp. (OM169327) was isolated from the soil as mentioned in the previous study carried out by Hajji-Hedfi et al. [14] by the dilution soil plate technique. The taxon was isolated on Czapek’s yeast extract agar (30 g/L sucrose; 3.0 g/L sodium nitrate; 0.5 g/L potassium chloride; 0.5 g/L magnesium sulfate heptahydrate; 0.01 g/L iron (II) sulfate heptahydrate; 1.0 g/L di-potassium hydrogen phosphate; 5.0 g/L yeast extract; 15.0 g/L agar agar) and potato dextrose agar (PDA). Both isolation media were supplemented with Rose Bengal (1/15,000) and chloramphenicol (50 ppm) for the suppression of bacterial growth [14]. The plates were incubated (BJPX-HTBII, Biobase, Jinan, China) at 25 ± 2 °C for 7 days, and then the developing colonies were identified. The fungus culture was maintained on PDA (Potato dextrose agar) for 10 days at 25 °C ± 2 °C. Morphological characterization was performed by macroscopic and microscopic observation according to the relevant identification key of Zare and Gams [47], followed by molecular confirmation. Total genomic DNA was extracted from mycelia scraped from the surface of the pure culture. Partial 28 S rDNA was amplified using primers ITS1/ITS4 according to the methods of White et al. [48]. The obtained sequence was submitted to Genbank and assigned the accession number: OM169327. Lecanicillium sp. (OM169327) was deposited in the Fungarium of Suez Canal University (https://ccinfo.wdcm.org/collection/by_id/1180), at Botany and Microbiology Department, Faculty of Science, Ismailia 41,522, Egypt under accession number SCUF 1010.

#### Extraction of Lecanicillium sp filtrate

Filtrate preparation was carried out by transferring the fungus to potato dextrose broth (BDB) to be incubated at 25 °C ± 2 °C for 15 days and then filtered through a Whatman filter paper N°1 to remove the mycelia mats followed by a 0.45 μm Millipore filter. The collected filtrate was used net (100%) or diluted in distilled water (10%, 25%, 50%, 75%; v/v). All filtrate concentrations were kept at 4 °C until use [49, 50].

Liquid suspensions of approximately 100 eggs (200 µl) and ± 20 juveniles (100 µl) were separately placed onto 5 cm Petri plates with 2 ml of various concentrations of fungal filtrate culture, and a distilled water control was added. The plates were incubated in darkness at 25 °C for seven days for the egg-hatching test and three days for the larval mortality test. Each treatment was replicated five times [49, 50].

For the juveniles’ assay, the immobile and mobile nematodes were counted under a stereomicroscope. The juveniles were then transferred to distilled water for 24 h to confirm death. The net mortality was calculated according to Abott’s formula. For the egg-hatching assay, the number of hatched juveniles was counted under a binocular microscope and the relative egg-hatching was determined. The bioassays were repeated twice, and the means of the two experiments were subjected to statistical analysis using the SPSS package (SPSS Statistics, v. 20 for Windows). All data were expressed as means ± SD. The level of significance was set at p < 0.05. Statistically significant values were analyzed using ANOVA with Tukey’s multiple comparison tests [49, 50].

### Phylogenetic analysis

Phylogenetic analysis was performed to confirm the isolate’s taxonomic status by determining the systematic placement and relationships with other closely related taxa. The analysed sequence of the *Lecanicillium* sp. was aligned against reference sequences of other *Lecanicillium* accessions using the online multiple alignment program for nucleotide sequences (MAFFT version 7). All reference sequences were obtained from the NCBI database. Aligned sequence data were transferred to the MEGA11 analysis program, where the evolutionary history was inferred using the UPGMA method [51, 52].

This analysis involved 17 nucleotide sequences. The bootstrap consensus tree inferred from 1000 replicates represents the evolutionary history of the analysed taxa. Branches corresponding to partitions reproduced in less than 50% of the bootstrap replicates are collapsed. The percentage of replicate trees in which the associated taxa are clustered together in the bootstrap test (1000 replicates) is shown next to the branches [51, 52].

### Proteome analysis

#### Extraction of fungal mycelia proteins

Produced mycelia of *Lecanicillium* sp. On BDB were crushed by a pestle in a 1.5 ml Eppendorf tube on ice and suspended in 200 µl of lysis buffer (7 M urea, 2 M thiourea, 2% (v/v) triton-100, IPG buffer pH 3–11, 2% (v/v), 40mM DTT with protease and phosphatase inhibitors). Total proteins were precipitated using the methanol/chloroform method [50]. Protein pellets were re-suspended and denatured in 7 M Urea/2 M Thiourea/100 mM TEAB, pH 7.5. Protein concentration was estimated using the RC DC Protein Assay kit (BIO-RAD) according to the manufacturer’s instructions [50].

#### Protein digestion

Cell pellets were dissolved in a lysis buffer (8 M urea, 2 M thiourea, 5% CHAPS, 2 mM TCEP-HCl and protease inhibitor). The cells were homogenized by ultra-sonication (10 strokes, low amplitude) on ice. After homogenization, the lysed cells were centrifuged at 20,000×g for 10 min at 4 °C, and the supernatant containing the solubilized proteins was used for analysis. Total protein concentration was determined using a Pierce 660 nm protein assay (Thermo). Prior to digestion, the total proteins from each sample were precipitated by the methanol/chloroform method [50].

For digestion, protein pellets were re-suspended and denatured in 20 µl 7 M Urea/2 M Thiourea/100 mM TEAB, pH 7.5, reduced with 2 µL of 50mMTris (2-carboxyethyl) phosphine (TCEP, MERCK), pH 8.0, at 37 °C for 60 min and followed by 2 µL of 200 mM cysteine-blocking reagent (methyl methane thiosulfonate [MMTS, Pierce]) for 10 min at room temperature. Samples were diluted up to 120 µL to reduce urea concentration with 25 mM TEAB. Digestions were initiated by adding 1 µL (1 µg/µL) sequence grade-modified trypsin (Pierce) to each sample, which was then incubated at 37 °C overnight on a shaker. Sample digestions were evaporated until dry and then desalted onto SEP-PAK C18 cartridge (Waters) until the mass spectrometric analysis [50].

#### Liquid chromatography and mass spectrometer analysis

Digested peptides of each sample were subjected to 1D-nano LC ESI-MSMS analysis using a nano liquid chromatography system (Eksigent Technologies nanoLC Ultra 1D plus, SCIEX, Foster City, CA) coupled to a high-speed Triple TOF 5600 mass spectrometer (SCIEX, Foster City, CA) with a Nanospray III Source. The analytical column used was a silica-based reversed-phase Acquity UPLC® M-Class Peptide BEH C18 Column (Waters) [48].

The trap column was a C18 Acclaim PepMap™ 100 (Thermo-Fisher Scientific Inc.), 100 μm × 2 cm, 5 μm particle diameter, 100 Å pore size, switched on-line with the analytical column. The loading pump delivered a solution of 0.1% formic acid in water at 2 µL/min. The nano-pump provided a flow rate of 300 mL/min and was operated under gradient elution conditions. Peptides were separated using a 250 min-gradient ranging from 2 to 90% mobile phase B (mobile phase A: 2% acetonitrile, 0.1% formic acid; mobile phase B: 100% acetonitrile, 0.1% formic acid). The injection volume was 5 µl [50].

#### Data acquisition and analysis

Data acquisition was performed with a Triple TOF 5600 System (SCIEX, Foster City, CA). Data were acquired using an IonSpray voltage floating (ISVF) 2800 V, curtain gas (CUR) 20, interface heater temperature (IHT) 150, ion source gas 1 (GS1) 20, declustering potential (DP) 85 V. All data were acquired using information-dependent acquisition (IDA) mode with Analyst TF 1.7 software (SCIEX, Foster City, CA). For IDA parameters, 0.25 s MS survey scan in the mass range of 350–1250 Da were followed by 35 MS/MS scans of 100ms in the mass range of 100–1800 (total cycle time: 3.8 s). Switching criteria were set to ions greater than the mass-to-charge ratio (m/z) 350 and smaller than m/z 1250 with a charge state of 2–5 and an abundance threshold of more than 90 counts (cps). Former target ions were excluded for 20 s. IDA rolling collision energy (CE) parameters script was used for automatically controlling the CE [50].

MS and MS/MS data obtained for individual samples were processed using Analyst® TF 1.7 Software (SCIEX, Foster City, CA). Raw data file conversion tools generated MGFfiles, which were also searched against the protein database, containing protein-coding genes and other common protein contaminants using the Mascot Server v. 2.6 (Matrix Science, London, UK). Search parameters were set as follows: methylthio(C) as fixed modification and oxidation(M) as variable modifications. Peptide mass tolerance was set to 25 ppm and 0.05 Da for fragment masses, also two missed cleavages were allowed. The confidence interval for protein identification was set to ≥ 95% (p < 0.05) and only peptides with an individual ion score above the 1% False Discovery Rates (FDR) at peptide level were considered correctly identified. [50].

### Statistical analysis

All data were subjected to analysis of variance and the means compared by Tukey’s multiple-range test. Differences at p ≤ 0.05 were considered significant. Statistical analysis was performed by using SPSS 20 Software for Windows.

## Electronic supplementary material

Below is the link to the electronic supplementary material.

Supplementary Material 1

## Data Availability

Sequence was submitted to Genbank and assigned the accession number: OM169327. *Lecanicillium* sp. (OM169327) was deposited in the Fungarium of Suez Canal University (https://ccinfo.wdcm.org/collection/by_id/1180), at Botany and Microbiology Department, Faculty of Science, Ismailia 41,522, Egypt under accession number SCUF 1010.
