# Supplementary material for: Biological and proteomic analysis of a new isolate of the nematophagous fungus lecanicillium sp
Source: BMC Microbiol. 2023 Apr 20;23:108. doi: 10.1186/s12866-023-02855-4 (PMC10116813; doi:10.1186/s12866-023-02855-4)
Supplement: Supplementary file 1 — Supplementary Material 1 [file 12866_2023_2855_MOESM1_ESM.docx]

**Supplementary file. Peptide list reflected the proteins with show at least two peptides as minimum criterion for identification**

| **Uniprot_Acc** | **Peptide Sequence/Num. Pept** | **Modifications** | **Pept. Score** | **m/z** | **Mr (Da)** | **z (Charge)** | **Delta (Da)** | **pep_expect** |
| --- | --- | --- | --- | --- | --- | --- | --- | --- |
| **P04264** | 62 |  |  |  |  |  |  |  |
|  | SGGGFSSGSAGIINYQR |  | 156,6 | 829,409 | 1656,803 | 2 | 0,017 | 6,00E-15 |
|  | FSSCGGGGGSFGAGGGFGSR |  | 155,79 | 877,864 | 1753,714 | 2 | 0,02 | 5,90E-15 |
|  | GSYGSGGSSYGSGGGSYGSGGGGGGHGSYGSGSSSGGYR | | 152,55 | 1104,777 | 3311,309 | 3 | 0,008 | 7,70E-14 |
|  | NKLNDLEDALQQAKEDLAR |  | 122,67 | 728,721 | 2183,141 | 3 | 0,022 | 7,40E-12 |
|  | GGGGGGYGSGGSSYGSGGGSYGSGGGGGGGR |  | 114,1 | 795,327 | 2382,96 | 3 | 0,015 | 1,30E-10 |
|  | QISNLQQSISDAEQR |  | 112,99 | 858,937 | 1715,86 | 2 | 0,016 | 3,30E-10 |
|  | MSGECAPNVSVSVSTSHTTISGGGSR | Oxidation (M) | 108,75 | 857,39 | 2569,149 | 3 | 0,028 | 6,40E-11 |
|  | LDNLQQEIDFLTALYQAELSQMQTQISETNVILSMDNNR | Oxidation (M) | 105,96 | 1136,818 | 4543,242 | 4 | 1,053 | 1,20E-10 |
|  | SLDLDSIIAEVK |  | 101,67 | 651,867 | 1301,72 | 2 | 0,012 | 2,40E-08 |
|  | LNDLEDALQQAKEDLAR |  | 99,99 | 648,006 | 1940,997 | 3 | 0,017 | 1,90E-09 |
|  | FLEQQNQVLQTK |  | 97,82 | 738,403 | 1474,791 | 2 | 0,013 | 2,20E-09 |
|  | SKAEAESLYQSKYEELQITAGR |  | 96,34 | 626,074 | 2500,266 | 4 | 0,022 | 3,30E-09 |
|  | TNAENEFVTIKK |  | 94,09 | 465,251 | 1392,732 | 3 | 0,007 | 5,70E-08 |
|  | THNLEPYFESFINNLR |  | 93,82 | 665,337 | 1992,989 | 3 | 0,02 | 8,20E-09 |
|  | NSKIEISELNR |  | 93,35 | 434,907 | 1301,698 | 3 | 0,005 | 2,00E-07 |
|  | KQISNLQQSISDAEQR |  | 92,72 | 615,656 | 1843,946 | 3 | 0,008 | 5,10E-09 |
|  | SLNNQFASFIDK |  | 92,23 | 692,354 | 1382,694 | 2 | 0,011 | 1,60E-08 |
|  | AEAESLYQSKYEELQITAGR |  | 92,22 | 762,721 | 2285,14 | 3 | 0,023 | 8,00E-09 |
|  | KDVDGAYMTK | Oxidation (M) | 92,13 | 572,274 | 1142,533 | 2 | 0,005 | 7,50E-08 |
|  | MSGECAPNVSVSVSTSHTTISGGGSR |  | 86,49 | 852,058 | 2553,154 | 3 | 0,028 | 7,80E-09 |
|  | LNDLEDALQQAK |  | 86,17 | 679,358 | 1356,702 | 2 | 0,013 | 9,90E-07 |
|  | LALDLEIATYR |  | 84,39 | 639,864 | 1277,712 | 2 | 1,01 | 1,30E-07 |
|  | SLNNQFASFIDKVR |  | 84,26 | 546,961 | 1637,861 | 3 | 0,009 | 1,80E-08 |
|  | TNAENEFVTIK |  | 81,35 | 633,327 | 1264,639 | 2 | 0,009 | 5,40E-07 |
|  | NKLNDLEDALQQAK |  | 79,79 | 533,953 | 1598,837 | 3 | 0,011 | 5,40E-07 |
|  | SKAEAESLYQSK |  | 77,97 | 670,841 | 1339,667 | 2 | 0,005 | 2,60E-06 |
|  | QISNLQQSISDAEQR | Gln->pyro-Glu (N-term Q) | 77,65 | 850,427 | 1698,839 | 2 | 0,022 | 1,30E-06 |
|  | WELLQQVDTSTR |  | 76,83 | 738,386 | 1474,758 | 2 | 0,016 | 7,90E-07 |
|  | YEELQITAGR |  | 73,99 | 590,307 | 1178,599 | 2 | 0,006 | 1,60E-05 |
|  | MSGECAPNVSVSVSTSHTTISGGGSR |  | 72,4 | 639,294 | 2553,148 | 4 | 0,022 | 1,60E-07 |
|  | AEAESLYQSK |  | 72,22 | 563,277 | 1124,54 | 2 | 0,005 | 6,70E-06 |
|  | LDSELKNMQDMVEDYR |  | 72,06 | 662,642 | 1984,905 | 3 | 0,018 | 1,80E-07 |
|  | QISNLQQSISDAEQRGENALK |  | 70,39 | 777,071 | 2328,192 | 3 | 0,025 | 4,70E-07 |
|  | NKYEDEINKR |  | 69,19 | 436,891 | 1307,651 | 3 | 0,004 | 1,60E-05 |
|  | QISNLQQSISDAEQR |  | 68,75 | 572,959 | 1715,854 | 3 | 0,01 | 8,10E-07 |
|  | SKAEAESLYQSK |  | 67,76 | 447,562 | 1339,665 | 3 | 0,004 | 3,20E-06 |
|  | TLLEGEESR |  | 67,68 | 517,264 | 1032,513 | 2 | 0,005 | 2,00E-05 |
|  | IEISELNR |  | 67,32 | 487,271 | 972,528 | 2 | 0,004 | 0,0001 |
|  | NMQDMVEDYR | Oxidation (M) | 66,97 | 658,77 | 1315,525 | 2 | 0,008 | 5,30E-07 |
|  | QISNLQQSISDAEQRGENALKDAK |  | 65,67 | 661,595 | 2642,35 | 4 | 0,024 | 7,00E-07 |
|  | NSKIEISELNR |  | 65,61 | 651,858 | 1301,701 | 2 | 0,007 | 0,0001 |
|  | WELLQQVDTSTR |  | 63,54 | 492,591 | 1474,751 | 3 | 0,01 | 1,80E-05 |
|  | THNLEPYFESFINNLR |  | 61,85 | 997,506 | 1992,998 | 2 | 0,029 | 2,50E-06 |
|  | FVSTTYSGVTR |  | 61,74 | 609,315 | 1216,616 | 2 | 0,007 | 1,70E-05 |
|  | SLVNLGGSK |  | 61,6 | 437,755 | 873,496 | 2 | 0,004 | 2,50E-05 |
|  | LDSELKNMQDMVEDYR | Oxidation (M) | 59,76 | 667,975 | 2000,904 | 3 | 0,022 | 2,50E-06 |
|  | NMQDMVEDYR |  | 59,31 | 650,773 | 1299,531 | 2 | 0,009 | 2,70E-06 |
|  | QISNLQQSISDAEQRGENALK | Gln->pyro-Glu (N-term Q) | 56,35 | 771,395 | 2311,163 | 3 | 0,023 | 5,20E-06 |
|  | SISISVAR |  | 56,31 | 416,75 | 831,484 | 2 | 0,003 | 0,00086 |
|  | QISNLQQSISDAEQRGENALKDAK | Gln->pyro-Glu (N-term Q) | 55,17 | 876,117 | 2625,328 | 3 | 0,028 | 6,70E-06 |
|  | QISNLQQSISDAEQRGENALKDAK |  | 54,7 | 881,793 | 2642,358 | 3 | 0,032 | 7,50E-06 |
|  | LDNLQQEIDFLTALYQAELSQMQTQISETNVILSMDNNR | | 54,01 | 1132,579 | 4526,289 | 4 | 0,094 | 8,60E-06 |
|  | AQYEDIAQK |  | 51,16 | 533,267 | 1064,519 | 2 | 0,005 | 0,00031 |
|  | NKLNDLEDALQQAKEDLAR |  | 50,94 | 546,791 | 2183,133 | 4 | 0,015 | 1,70E-05 |
|  | DYQELMNTK | Oxidation (M) | 50,83 | 579,262 | 1156,509 | 2 | 0,002 | 1,70E-05 |
|  | DVDGAYMTK | Oxidation (M) | 49,91 | 508,226 | 1014,437 | 2 | 0,004 | 3,20E-05 |
|  | LRSEIDNVKK |  | 49,84 | 401,236 | 1200,687 | 3 | 0,004 | 0,0032 |
|  | FSSCGGGGGSFGAGGGFGSR |  | 49,82 | 585,576 | 1753,706 | 3 | 0,012 | 2,10E-05 |
|  | LDSELKNMQDMVEDYR |  | 49,65 | 993,466 | 1984,917 | 2 | 0,03 | 2,50E-05 |
|  | TNAENEFVTIKK |  | 48,9 | 697,864 | 1393,713 | 2 | 0,988 | 4,20E-05 |
|  | SGGGFSSGSAGIINYQR |  | 48,86 | 553,272 | 1656,796 | 3 | 0,01 | 2,80E-05 |
|  | DYQELMNTK |  | 48,61 | 571,266 | 1140,517 | 2 | 0,004 | 2,80E-05 |
| **P13645** | 48 |  |  |  |  |  |  |  |
|  | SGGGGGGGGCGGGGGVSSLR |  | 207,29 | 769,83 | 1537,645 | 2 | 0,009 | 1,40E-19 |
|  | GSLGGGFSSGGFSGGSFSR |  | 150,9 | 854,399 | 1706,784 | 2 | 0,02 | 4,70E-14 |
|  | HYSSSRSGGGGGGGGCGGGGGVSSLR |  | 144,29 | 752,99 | 2255,95 | 3 | 0,994 | 3,00E-14 |
|  | TIDDLKNQILNLTTDNANILLQIDNAR |  | 143,62 | 1018,231 | 3051,671 | 3 | 0,051 | 4,50E-14 |
|  | NQILNLTTDNANILLQIDNAR |  | 138,19 | 789,768 | 2366,281 | 3 | 0,026 | 4,30E-13 |
|  | GSSGGGCFGGSSGGYGGLGGFGGGSFR |  | 129,41 | 1166,492 | 2330,97 | 2 | 0,027 | 7,40E-13 |
|  | SKELTTEIDNNIEQISSYK |  | 126,9 | 738,046 | 2211,115 | 3 | 0,024 | 2,20E-12 |
|  | YCVQLSQIQAQISALEEQLQQIR |  | 124,46 | 912,479 | 2734,416 | 3 | 0,038 | 2,10E-12 |
|  | NVSTGDVNVEMNAAPGVDLTQLLNNMR |  | 119,53 | 958,15 | 2871,427 | 3 | 0,041 | 1,30E-10 |
|  | QSLEASLAETEGR |  | 115,72 | 695,849 | 1389,684 | 2 | 0,01 | 3,50E-10 |
|  | NVQALEIELQSQLALK |  | 113,63 | 599,681 | 1796,02 | 3 | 0,016 | 9,70E-11 |
|  | GSLGGGFSSGGFSGGSFSRGSSGGGCFGGSSGGYGGLGGFGGGSFR | | 108,79 | 1006,189 | 4020,727 | 4 | 1,029 | 6,30E-11 |
|  | ADLEMQIESLTEELAYLK |  | 108,13 | 1048,544 | 2095,072 | 2 | 0,033 | 1,60E-10 |
|  | GSSGGGCFGGSSGGYGGLGGFGGGSFR |  | 100,76 | 777,995 | 2330,963 | 3 | 0,019 | 3,60E-10 |
|  | NVQALEIELQSQLALK |  | 99,43 | 899,021 | 1796,028 | 2 | 0,024 | 2,40E-09 |
|  | ADLEMQIESLTEELAYLKK | Oxidation (M) | 97,67 | 747,393 | 2239,158 | 3 | 0,029 | 7,00E-10 |
|  | SLLEGEGSSGGGGR |  | 95,79 | 631,805 | 1261,596 | 2 | 0,006 | 8,00E-09 |
|  | QSLEASLAETEGR | Gln->pyro-Glu (N-term Q) | 95,17 | 687,337 | 1372,659 | 2 | 0,012 | 8,40E-09 |
|  | NVSTGDVNVEMNAAPGVDLTQLLNNMR | Oxidation (M) | 94,17 | 963,481 | 2887,42 | 3 | 0,039 | 1,50E-09 |
|  | ADLEMQIESLTEELAYLKK |  | 93,56 | 742,061 | 2223,162 | 3 | 0,027 | 1,70E-09 |
|  | QSVEADINGLR |  | 92,7 | 601,316 | 1200,617 | 2 | 0,007 | 5,90E-08 |
|  | VTMQNLNDR | Oxidation (M) | 92,52 | 553,768 | 1105,521 | 2 | 0,003 | 1,70E-07 |
|  | ELTTEIDNNIEQISSYK |  | 91,16 | 999,496 | 1996,978 | 2 | 1,015 | 1,80E-08 |
|  | ELTTEIDNNIEQISSYK |  | 91,06 | 666,334 | 1995,981 | 3 | 0,018 | 2,10E-07 |
|  | ALEESNYELEGK |  | 87,39 | 691,332 | 1380,65 | 2 | 0,01 | 1,90E-08 |
|  | AETECQNTEYQQLLDIK |  | 85,45 | 691,321 | 2070,941 | 3 | 0,018 | 9,70E-09 |
|  | ADLEMQIESLTEELAYLK | Oxidation (M) | 82,57 | 1056,543 | 2111,071 | 2 | 0,037 | 1,80E-08 |
|  | RNVQALEIELQSQLALK |  | 79,01 | 651,713 | 1952,118 | 3 | 0,012 | 8,30E-07 |
|  | LENEIQTYR |  | 76,59 | 583,298 | 1164,582 | 2 | 0,005 | 1,10E-06 |
|  | VLDELTLTKADLEMQIESLTEELAYLK |  | 76,04 | 1036,906 | 3107,697 | 3 | 0,077 | 7,40E-08 |
|  | VLDELTLTK |  | 72,47 | 516,306 | 1030,598 | 2 | 0,006 | 1,80E-05 |
|  | SKELTTEIDNNIEQISSYKSEITELR |  | 72,24 | 760,895 | 3039,553 | 4 | 0,028 | 1,70E-07 |
|  | SQYEQLAEQNRK |  | 69,55 | 498,585 | 1492,732 | 3 | 0,005 | 7,30E-07 |
|  | LKYENEVALR |  | 68,75 | 412,233 | 1233,677 | 3 | 0,005 | 7,30E-06 |
|  | VTMQNLNDR |  | 67,43 | 545,771 | 1089,528 | 2 | 0,004 | 1,10E-05 |
|  | SQYEQLAEQNR |  | 62,9 | 683,326 | 1364,638 | 2 | 0,006 | 1,80E-06 |
|  | NHEEEMKDLR | Oxidation (M) | 59,88 | 439,536 | 1315,586 | 3 | 0,003 | 7,00E-06 |
|  | RVLDELTLTK |  | 58,55 | 594,357 | 1186,7 | 2 | 0,008 | 0,00028 |
|  | IRLENEIQTYR |  | 57,86 | 478,93 | 1433,769 | 3 | 0,006 | 0,00025 |
|  | IRLENEIQTYR |  | 55,22 | 717,894 | 1433,773 | 2 | 0,01 | 2,60E-05 |
|  | LASYLDKVR |  | 54,46 | 532,811 | 1063,608 | 2 | 0,005 | 0,00051 |
|  | VLDELTLTKADLEMQIESLTEELAYLKK |  | 53,46 | 809,948 | 3235,764 | 4 | 0,049 | 9,70E-06 |
|  | LASYLDKVR |  | 51,72 | 355,542 | 1063,605 | 3 | 0,002 | 0,0014 |
|  | NHEEEMKDLR |  | 50,75 | 434,205 | 1299,593 | 3 | 0,005 | 3,20E-05 |
|  | DAEAWFNEK |  | 49,44 | 555,252 | 1108,49 | 2 | 0,008 | 0,0002 |
|  | LAADDFR |  | 49,18 | 404,204 | 806,394 | 2 | 0,002 | 0,0041 |
|  | YENEVALR |  | 46,95 | 497,256 | 992,496 | 2 | 0,004 | 0,0077 |
|  | IKEWYEK |  | 45,52 | 498,265 | 994,516 | 2 | 0,004 | 0,0052 |
| **P35908** | 47 |  |  |  |  |  |  |  |
|  | GGSGGGGSISGGGYGSGGGSGGR |  | 154,39 | 871,382 | 1740,749 | 2 | 0,008 | 4,20E-14 |
|  | GSSSGGGYSSGSSSYGSGGR |  | 149,26 | 870,86 | 1739,705 | 2 | 0,007 | 6,00E-14 |
|  | GGGFGGGSSFGGGSGFSGGGFGGGGFGGGR |  | 124,12 | 800,353 | 2398,038 | 3 | 0,026 | 2,50E-12 |
|  | SISISVAGGGGGFGAAGGFGGR |  | 123,19 | 919,972 | 1837,929 | 2 | 0,022 | 2,80E-12 |
|  | VLYDAEISQIHQSVTDTNVILSMDNSR |  | 118,19 | 1016,849 | 3047,526 | 3 | 0,039 | 8,30E-12 |
|  | NKLNDLEEALQQAKEDLAR |  | 109,61 | 733,392 | 2197,154 | 3 | 0,02 | 3,90E-10 |
|  | VDLLNQEIEFLK |  | 105,48 | 730,911 | 1459,808 | 2 | 0,015 | 1,80E-09 |
|  | FLEQQNQVLQTK |  | 97,82 | 738,403 | 1474,791 | 2 | 0,013 | 2,20E-09 |
|  | MSGDLSSNVTVSVTSSTISSNVASK | Oxidation (M) | 96,76 | 825,41 | 2473,209 | 3 | 0,023 | 8,50E-10 |
|  | TSQNSELNNMQDLVEDYKK | Oxidation (M) | 93,92 | 758,023 | 2271,047 | 3 | 0,015 | 2,00E-09 |
|  | MSGDLSSNVTVSVTSSTISSNVASK |  | 91,4 | 820,08 | 2457,217 | 3 | 0,027 | 2,70E-09 |
|  | TAAENDFVTLK |  | 90,79 | 604,815 | 1207,616 | 2 | 0,008 | 1,20E-08 |
|  | SKEEAEALYHSKYEELQVTVGR |  | 85,48 | 642,33 | 2565,29 | 4 | 0,019 | 2,50E-08 |
|  | AAFGGSGGRGSSSGGGYSSGSSSYGSGGR |  | 85,47 | 834,694 | 2501,06 | 3 | 1 | 9,70E-09 |
|  | GFSSGSAVVSGGSR |  | 81,84 | 627,809 | 1253,604 | 2 | 0,004 | 1,30E-07 |
|  | NLDLDSIIAEVK |  | 81,79 | 665,377 | 1328,739 | 2 | 0,02 | 2,50E-07 |
|  | LNDLEEALQQAKEDLAR |  | 81,17 | 652,678 | 1955,012 | 3 | 0,016 | 2,70E-07 |
|  | TSQNSELNNMQDLVEDYKK |  | 80,18 | 752,695 | 2255,062 | 3 | 0,025 | 3,40E-08 |
|  | LALDVEIATYR |  | 80,17 | 632,356 | 1262,697 | 2 | 0,01 | 3,00E-06 |
|  | LNDLEEALQQAK |  | 78,8 | 686,365 | 1370,716 | 2 | 0,012 | 1,30E-07 |
|  | NVQDAIADAEQR |  | 76,8 | 665,325 | 1328,636 | 2 | 0,004 | 1,70E-06 |
|  | YEELQVTVGR |  | 74,7 | 597,315 | 1192,616 | 2 | 0,008 | 1,40E-05 |
|  | KLLEGEECR |  | 71,05 | 561,769 | 1121,523 | 2 | 0,002 | 3,50E-07 |
|  | QSGSRGGSGGGGSISGGGYGSGGGSGGR |  | 67,51 | 753,331 | 2256,972 | 3 | 0,986 | 8,60E-07 |
|  | IEISELNR |  | 67,32 | 487,271 | 972,528 | 2 | 0,004 | 0,0001 |
|  | GSSGEAFGSSVTFSFR |  | 66,39 | 811,886 | 1621,757 | 2 | 0,02 | 4,50E-06 |
|  | SKEEAEALYHSK |  | 63,96 | 464,566 | 1390,678 | 3 | 0,005 | 7,90E-06 |
|  | LQGEIAHVK |  | 62,18 | 497,79 | 993,565 | 2 | 0,004 | 1,70E-05 |
|  | NKLNDLEEALQQAKEDLAR |  | 61,69 | 550,294 | 2197,148 | 4 | 0,015 | 7,90E-05 |
|  | VLYDAEISQIHQSVTDTNVILSMDNSR | Oxidation (M) | 61,61 | 1022,179 | 3063,516 | 3 | 0,034 | 1,70E-06 |
|  | HGDSLKEIKIEISELNR |  | 61,28 | 496,025 | 1980,072 | 4 | 0,009 | 8,50E-06 |
|  | LLEGEECR |  | 61,1 | 497,722 | 993,43 | 2 | 0,004 | 2,30E-05 |
|  | AQYEEIAQR |  | 58,97 | 554,277 | 1106,539 | 2 | 0,003 | 0,00014 |
|  | LALDVEIATYRK |  | 58,75 | 696,404 | 1390,794 | 2 | 0,012 | 1,20E-05 |
|  | SLVGLGGTK |  | 57,73 | 416,252 | 830,489 | 2 | 0,003 | 0,00021 |
|  | YLDGLTAER |  | 57,06 | 519,269 | 1036,524 | 2 | 0,005 | 0,00062 |
|  | HGGGGGGFGGGGFGSR |  | 55,41 | 440,867 | 1319,579 | 3 | 0,003 | 0,0001 |
|  | TAAENDFVTLKK |  | 54,99 | 446,244 | 1335,709 | 3 | 0,006 | 1,00E-05 |
|  | FASFIDKVR |  | 54,47 | 541,806 | 1081,597 | 2 | 0,005 | 8,90E-05 |
|  | FASFIDKVR |  | 54,05 | 361,539 | 1081,595 | 3 | 0,003 | 0,00016 |
|  | STSSFSCLSR |  | 52,97 | 560,745 | 1119,475 | 2 | 0,006 | 0,00036 |
|  | LALDVEIATYR |  | 50,11 | 421,904 | 1262,691 | 3 | 0,004 | 0,0034 |
|  | DVDNAYMIK | Oxidation (M) | 49,56 | 542,754 | 1083,494 | 2 | 0,004 | 2,30E-05 |
|  | WELLQQMNVGTRPINLEPIFQGYIDSLKR | Oxidation (M) | 49,36 | 869,724 | 3474,866 | 4 | 1,053 | 2,40E-05 |
|  | LALDVEIATYRK |  | 48,03 | 464,603 | 1390,788 | 3 | 0,006 | 0,00015 |
|  | FGGFGGPGGVGGLGGPGGFGPGGYPGGIHEVSVNQSLLQPLNVK | | 47,08 | 1023,788 | 4091,123 | 4 | 0,058 | 3,80E-05 |
|  | FASFIDK |  | 46,78 | 414,22 | 826,426 | 2 | 0,003 | 0,0052 |
| **P35527** | 46 |  |  |  |  |  |  |  |
|  | GGSGGSHGGGSGFGGESGGSYGGGEEASGSGGGYGGGSGK | | 207,7 | 1075,106 | 3222,297 | 3 | 0,023 | 3,40E-20 |
|  | GGSGGSYGGGGSGGGYGGGSGSR |  | 175,14 | 896,373 | 1790,731 | 2 | 0,01 | 1,60E-16 |
|  | SGGGGGGGLGSGGSIR |  | 131,91 | 616,805 | 1231,595 | 2 | 0,005 | 2,20E-11 |
|  | GGGGSFGYSYGGGSGGGFSASSLGGGFGGGSR |  | 119,23 | 902,404 | 2704,191 | 3 | 0,037 | 1,20E-10 |
|  | YCGQLQMIQEQISNLEAQITDVR |  | 115,37 | 909,452 | 2725,334 | 3 | 0,047 | 2,70E-11 |
|  | LASYLDKVQALEEANNDLENK |  | 112,92 | 793,075 | 2376,205 | 3 | 0,024 | 2,60E-11 |
|  | NYSPYYNTIDDLKDQIVDLTVGNNK |  | 104,74 | 968,155 | 2901,444 | 3 | 0,041 | 1,50E-10 |
|  | GGSGGSYGGGGSGGGYGGGSGSR |  | 103,38 | 597,916 | 1790,727 | 3 | 0,007 | 2,30E-09 |
|  | EIETYHNLLEGGQEDFESSGAGK |  | 103,11 | 837,392 | 2509,154 | 3 | 0,029 | 9,30E-10 |
|  | HGVQELEIELQSQLSK |  | 102,01 | 919,497 | 1836,98 | 2 | 0,022 | 1,60E-09 |
|  | HGVQELEIELQSQLSK |  | 100,19 | 613,331 | 1836,971 | 3 | 0,013 | 1,40E-08 |
|  | VQALEEANNDLENK |  | 89,47 | 793,893 | 1585,771 | 2 | 0,012 | 4,10E-07 |
|  | DIENQYETQITQIEHEVSSSGQEVQSSAK |  | 87,81 | 1088,858 | 3263,551 | 3 | 0,044 | 5,80E-09 |
|  | SDLEMQYETLQEELMALKK |  | 86,74 | 767,053 | 2298,137 | 3 | 0,025 | 7,40E-09 |
|  | NHKEEMSQLTGQNSGDVNVEINVAPGK | Oxidation (M) | 85,39 | 728,608 | 2910,405 | 4 | 0,027 | 9,90E-09 |
|  | QGVDADINGLR |  | 83,04 | 579,302 | 1156,59 | 2 | 0,006 | 7,70E-07 |
|  | FSSSSGYGGGSSR |  | 80,01 | 618,27 | 1234,526 | 2 | 0,005 | 9,10E-07 |
|  | STMQELNSR | Oxidation (M) | 77,73 | 541,251 | 1080,488 | 2 | 0,001 | 1,50E-06 |
|  | HGVQELEIELQSQLSKK |  | 77,2 | 656,031 | 1965,071 | 3 | 0,018 | 3,00E-07 |
|  | QEIECQNQEYSLLLSIK |  | 74,42 | 695,68 | 2084,019 | 3 | 1,023 | 1,10E-07 |
|  | YCGQLQMIQEQISNLEAQITDVR | Oxidation (M) | 72,64 | 914,781 | 2741,322 | 3 | 0,04 | 1,50E-07 |
|  | QEYEQLIAK |  | 70,36 | 561,298 | 1120,582 | 2 | 0,006 | 7,80E-06 |
|  | TLLDIDNTR |  | 70,09 | 530,788 | 1059,562 | 2 | 0,006 | 2,70E-05 |
|  | IKFEMEQNLR | Oxidation (M) | 69,46 | 441,898 | 1322,671 | 3 | 0,006 | 4,50E-05 |
|  | NHKEEMSQLTGQNSGDVNVEINVAPGKDLTK |  | 68,53 | 838,926 | 3351,673 | 4 | 0,037 | 4,60E-07 |
|  | IKFEMEQNLR |  | 63,34 | 436,566 | 1306,676 | 3 | 0,006 | 5,00E-05 |
|  | QVLDNLTMEK |  | 62,4 | 596,317 | 1190,62 | 2 | 1,018 | 1,60E-05 |
|  | GFGGASGGGYSSSGGFGGGFGGGSGGGFGGGYGSGFGGFGGFGGGAGGGDGGILTANEK | | 62,25 | 1216,296 | 4861,154 | 4 | 0,081 | 1,60E-06 |
|  | QVLDNLTMEK | Oxidation (M) | 62,11 | 603,806 | 1205,598 | 2 | 0,002 | 7,20E-06 |
|  | QVLDNLTMEK | Gln->pyro-Glu (N-term Q) | 60,23 | 587,299 | 1172,584 | 2 | 0,009 | 5,70E-06 |
|  | NHKEEMSQLTGQNSGDVNVEINVAPGK |  | 58,93 | 724,609 | 2894,408 | 4 | 0,025 | 1,50E-05 |
|  | NHKEEMSQLTGQNSGDVNVEINVAPGKDLTK | Oxidation (M) | 57,72 | 842,924 | 3367,665 | 4 | 0,034 | 4,00E-06 |
|  | QVLDNLTMEKSDLEMQYETLQEELMALKK |  | 57,45 | 868,443 | 3469,744 | 4 | 0,041 | 4,10E-06 |
|  | QVLDNLTMEKSDLEMQYETLQEELMALKK | Gln->pyro-Glu (N-term Q) | 55,09 | 864,188 | 3452,722 | 4 | 0,046 | 6,80E-06 |
|  | IKFEMEQNLR | Oxidation (M) | 54,92 | 662,344 | 1322,674 | 2 | 0,009 | 7,40E-05 |
|  | VQALEEANNDLENKIQDWYDK |  | 54,25 | 845,749 | 2534,226 | 3 | 0,033 | 1,70E-05 |
|  | STMQELNSR |  | 52,24 | 533,255 | 1064,495 | 2 | 0,003 | 0,00017 |
|  | QGVDADINGLR | Gln->pyro-Glu (N-term Q) | 51,8 | 570,791 | 1139,567 | 2 | 0,01 | 6,60E-05 |
|  | QEIECQNQEYSLLLSIK |  | 48,93 | 1043,024 | 2084,033 | 2 | 1,037 | 2,60E-05 |
|  | EIETYHNLLEGGQEDFESSGAGK | Glu->pyro-Glu (N-term E) | 48,45 | 831,389 | 2491,144 | 3 | 0,03 | 3,20E-05 |
|  | IKFEMEQNLR |  | 48,39 | 654,347 | 1306,679 | 2 | 0,009 | 0,00047 |
|  | GGSGGSYGGGSGSGGGSGGGYGGGSGGGHSGGSGGGHSGGSGGNYGGGSGSGGGSGGGYGGGSGSR | | 48,38 | 1244,252 | 4972,978 | 4 | 1,049 | 0,0013 |
|  | MTLDDFR |  | 46,93 | 449,214 | 896,413 | 2 | 0,007 | 0,00013 |
|  | IQDWYDK |  | 46,6 | 484,233 | 966,451 | 2 | 0,007 | 0,0002 |
|  | MTLDDFR | Oxidation (M) | 45,94 | 457,21 | 912,405 | 2 | 0,004 | 0,00039 |
|  | QFSSSYLSR | Gln->pyro-Glu (N-term Q) | 45,55 | 529,254 | 1056,494 | 2 | 0,006 | 0,0022 |
| **P02534** | 13 |  |  |  |  |  |  |  |
|  | DSLENTLTETEAR |  | 94,52 | 739,859 | 1477,703 | 2 | 0,013 | 1,30E-08 |
|  | QNQEYQVLLDVR |  | 89,2 | 752,9 | 1503,785 | 2 | 0,017 | 6,50E-08 |
|  | SQLGDRLNVEVDAAPTVDLNR |  | 78,53 | 761,405 | 2281,192 | 3 | 0,026 | 3,30E-07 |
|  | TVNALEVELQAQHNLR |  | 75,42 | 612,335 | 1833,983 | 3 | 0,014 | 8,70E-08 |
|  | QLVESDINGLR |  | 74,1 | 622,34 | 1242,666 | 2 | 0,009 | 8,20E-06 |
|  | TVNALEVELQAQHNLRDSLENTLTETEAR |  | 74,07 | 824,429 | 3293,686 | 4 | 0,037 | 1,10E-07 |
|  | SQQQEPLVCPNYQSYFR |  | 70,84 | 711,661 | 2131,961 | 3 | 0,015 | 2,30E-07 |
|  | LNVEVDAAPTVDLNR |  | 68,71 | 813,437 | 1624,86 | 2 | 0,018 | 1,70E-06 |
|  | AQYEALVETNR |  | 58,96 | 647,328 | 1292,642 | 2 | 0,006 | 5,40E-05 |
|  | LVVQIDNAK |  | 51,36 | 500,297 | 998,58 | 2 | 0,004 | 0,00019 |
|  | LAADDFR |  | 49,18 | 404,204 | 806,394 | 2 | 0,002 | 0,0041 |
|  | QNQEYQVLLDVR | Gln->pyro-Glu (N-term Q) | 45,82 | 744,383 | 1486,752 | 2 | 0,01 | 7,70E-05 |
|  | SNHEEEVNTLR |  | 45,51 | 664,306 | 1326,597 | 2 | -0,02 | 5,60E-05 |
| **EEY14051.1** | 11 |  |  |  |  |  |  |  |
|  | SYELPDGQVITIGNER |  | 108,51 | 895,96 | 1789,906 | 2 | 0,022 | 5,70E-10 |
|  | QEYDESGPSIVHR | Gln->pyro-Glu (N-term Q) | 91,46 | 750,347 | 1498,678 | 2 | 0,01 | 2,70E-09 |
|  | DSYVGDEAQSKR |  | 80,3 | 678,319 | 1354,624 | 2 | 1,008 | 7,80E-08 |
|  | QEYDESGPSIVHR |  | 78,82 | 506,241 | 1515,701 | 3 | 0,006 | 4,10E-08 |
|  | IWHHTFYNELR |  | 73,88 | 505,924 | 1514,75 | 3 | 0,008 | 4,20E-07 |
|  | AGFAGDDAPR |  | 73,08 | 488,73 | 975,445 | 2 | 0,004 | 1,10E-05 |
|  | AVFPSIVGRPR |  | 72,62 | 400,242 | 1197,703 | 3 | 0,005 | 1,30E-06 |
|  | QEYDESGPSIVHRK |  | 71,07 | 548,939 | 1643,795 | 3 | 0,005 | 2,20E-07 |
|  | VAPEEHPVLLTEAPINPK |  | 69,47 | 652,033 | 1953,077 | 3 | 0,02 | 4,50E-07 |
|  | DSYVGDEAQSK |  | 56,5 | 599,766 | 1197,518 | 2 | 0,003 | 1,60E-05 |
|  | QEYDESGPSIVHRK | Gln->pyro-Glu (N-term Q) | 50,97 | 543,267 | 1626,778 | 3 | 0,014 | 1,70E-05 |
| **P25690** | 11 |  |  |  |  |  |  |  |
|  | DSLENTLTETEAR |  | 94,52 | 739,859 | 1477,703 | 2 | 0,013 | 1,30E-08 |
|  | QNQEYQVLLDVR |  | 89,2 | 752,9 | 1503,785 | 2 | 0,017 | 6,50E-08 |
|  | TVNALEVELQAQHNLR |  | 75,42 | 612,335 | 1833,983 | 3 | 0,014 | 8,70E-08 |
|  | TVNALEVELQAQHNLRDSLENTLTETEAR |  | 74,07 | 824,429 | 3293,686 | 4 | 0,037 | 1,10E-07 |
|  | SQQQEPLVCPNYQSYFR |  | 70,84 | 711,661 | 2131,961 | 3 | 0,015 | 2,30E-07 |
|  | AQYEALVETNR |  | 58,96 | 647,328 | 1292,642 | 2 | 0,006 | 5,40E-05 |
|  | QNHEQEVNTLR |  | 58,63 | 456,561 | 1366,662 | 3 | 0,003 | 2,40E-05 |
|  | LNVEVDAAPTVDLNHVLNETR |  | 56,38 | 774,083 | 2319,226 | 3 | 1,039 | 5,20E-06 |
|  | LVVQIDNAK |  | 51,36 | 500,297 | 998,58 | 2 | 0,004 | 0,00019 |
|  | SQLGDRLNVEVDAAPTVDLNHVLNETR |  | 46,22 | 744,64 | 2974,532 | 4 | 0,021 | 4,60E-05 |
|  | QNQEYQVLLDVR | Gln->pyro-Glu (N-term Q) | 45,82 | 744,383 | 1486,752 | 2 | 0,01 | 7,70E-05 |
| **P25691** | 9 |  |  |  |  |  |  |  |
|  | LAGLEEALQK |  | 71,91 | 536,811 | 1071,608 | 2 | 1,011 | 2,70E-05 |
|  | LLEGEEQR |  | 66,69 | 487,253 | 972,491 | 2 | 0,003 | 3,10E-05 |
|  | LTAEIENAK |  | 60,36 | 494,77 | 987,526 | 2 | 0,003 | 7,70E-05 |
|  | LGLDIEIATYR |  | 60,07 | 632,857 | 1263,7 | 2 | 1,013 | 0,00026 |
|  | ISPGYSVTR |  | 56,5 | 490,266 | 978,517 | 2 | 0,004 | 0,00013 |
|  | TKLEAAVAEAEQQGEAALNDAR |  | 54,86 | 762,391 | 2284,15 | 3 | 0,021 | 7,20E-06 |
|  | GGVACGGLTYSSTAGR |  | 52,87 | 752,35 | 1502,686 | 2 | 1,02 | 1,30E-05 |
|  | TKEEINELNR |  | 47,92 | 623,327 | 1244,64 | 2 | 0,004 | 0,00054 |
|  | AQYDDIASR |  | 46,72 | 519,749 | 1037,482 | 2 | 0,005 | 0,00053 |
| **P15241** | 8 |  |  |  |  |  |  |  |
|  | ATAENEFVALKK |  | 94,36 | 440,912 | 1319,713 | 3 | 0,004 | 2,30E-08 |
|  | LAGLEEALQK |  | 71,91 | 536,811 | 1071,608 | 2 | 1,011 | 2,70E-05 |
|  | LLEGEEQR |  | 66,69 | 487,253 | 972,491 | 2 | 0,003 | 3,10E-05 |
|  | LTAEVENAK |  | 60,83 | 487,761 | 973,508 | 2 | 0 | 3,30E-05 |
|  | LGLDIEIATYR |  | 60,07 | 632,857 | 1263,7 | 2 | 1,013 | 0,00026 |
|  | TKEEINELNR |  | 47,92 | 623,327 | 1244,64 | 2 | 0,004 | 0,00054 |
|  | AQYDDIASR |  | 46,72 | 519,749 | 1037,482 | 2 | 0,005 | 0,00053 |
|  | EAECVEADSGR |  | 46,63 | 606,238 | 1210,462 | 2 | 0,003 | 0,00027 |
| **CRK20212.1** | 6 |  |  |  |  |  |  |  |
|  | TVTSLDVVYALK |  | 104,88 | 654,879 | 1307,743 | 2 | 0,01 | 2,30E-09 |
|  | TVTSLDVVYALKR |  | 77 | 488,954 | 1463,841 | 3 | 0,006 | 6,00E-08 |
|  | ISAMIYEETR | Oxidation (M) | 76,51 | 614,804 | 1227,594 | 2 | 0,013 | 1,20E-07 |
|  | ISAMIYEETR |  | 70,91 | 606,805 | 1211,595 | 2 | 0,009 | 1,20E-06 |
|  | DNIQGITKPAIR |  | 63,45 | 442,591 | 1324,752 | 3 | 0,005 | 3,70E-05 |
|  | DAVTYTEHAK |  | 53,17 | 567,776 | 1133,538 | 2 | 0,003 | 6,40E-05 |
| **CRK28143.1** | 6 |  |  |  |  |  |  |  |
|  | TVTSLDVVYALK |  | 104,88 | 654,879 | 1307,743 | 2 | 0,01 | 2,30E-09 |
|  | TVTSLDVVYALKR |  | 77 | 488,954 | 1463,841 | 3 | 0,006 | 6,00E-08 |
|  | ISAMIYEETR | Oxidation (M) | 76,51 | 614,804 | 1227,594 | 2 | 0,013 | 1,20E-07 |
|  | ISAMIYEETR |  | 70,91 | 606,805 | 1211,595 | 2 | 0,009 | 1,20E-06 |
|  | DNIQGITKPAIR |  | 63,45 | 442,591 | 1324,752 | 3 | 0,005 | 3,70E-05 |
|  | DAVTYTEHAK |  | 53,17 | 567,776 | 1133,538 | 2 | 0,003 | 6,40E-05 |
| **O43790** | 6 |  |  |  |  |  |  |  |
|  | ATAENEFVALKK |  | 94,36 | 440,912 | 1319,713 | 3 | 0,004 | 2,30E-08 |
|  | LLEGEEQR |  | 66,69 | 487,253 | 972,491 | 2 | 0,003 | 3,10E-05 |
|  | LTAEVENAK |  | 60,83 | 487,761 | 973,508 | 2 | 0 | 3,30E-05 |
|  | LGLDIEIATYR |  | 60,07 | 632,857 | 1263,7 | 2 | 1,013 | 0,00026 |
|  | TKEEINELNR |  | 47,92 | 623,327 | 1244,64 | 2 | 0,004 | 0,00054 |
|  | EAECVEADSGR |  | 46,63 | 606,238 | 1210,462 | 2 | 0,003 | 0,00027 |
| **P78385** | 6 |  |  |  |  |  |  |  |
|  | ATAENEFVALKK |  | 94,36 | 440,912 | 1319,713 | 3 | 0,004 | 2,30E-08 |
|  | LLEGEEQR |  | 66,69 | 487,253 | 972,491 | 2 | 0,003 | 3,10E-05 |
|  | LTAEVENAK |  | 60,83 | 487,761 | 973,508 | 2 | 0 | 3,30E-05 |
|  | LGLDIEIATYR |  | 60,07 | 632,857 | 1263,7 | 2 | 1,013 | 0,00026 |
|  | TKEEINELNR |  | 47,92 | 623,327 | 1244,64 | 2 | 0,004 | 0,00054 |
|  | EAECVEADSGR |  | 46,63 | 606,238 | 1210,462 | 2 | 0,003 | 0,00027 |
| **Q15323** | 6 |  |  |  |  |  |  |  |
|  | QNQEYQVLLDVR |  | 89,2 | 752,9 | 1503,785 | 2 | 0,017 | 6,50E-08 |
|  | QLVESDINGLR |  | 74,1 | 622,34 | 1242,666 | 2 | 0,009 | 8,20E-06 |
|  | LNVEVDAAPTVDLNR |  | 68,71 | 813,437 | 1624,86 | 2 | 0,018 | 1,70E-06 |
|  | LVVQIDNAK |  | 51,36 | 500,297 | 998,58 | 2 | 0,004 | 0,00019 |
|  | LAADDFR |  | 49,18 | 404,204 | 806,394 | 2 | 0,002 | 0,0041 |
|  | QNQEYQVLLDVR | Gln->pyro-Glu (N-term Q) | 45,82 | 744,383 | 1486,752 | 2 | 0,01 | 7,70E-05 |
| **O76009** | 5 |  |  |  |  |  |  |  |
|  | QNQEYQVLLDVR |  | 89,2 | 752,9 | 1503,785 | 2 | 0,017 | 6,50E-08 |
|  | QLVESDINGLR |  | 74,1 | 622,34 | 1242,666 | 2 | 0,009 | 8,20E-06 |
|  | QNHEQEVNTLR |  | 58,63 | 456,561 | 1366,662 | 3 | 0,003 | 2,40E-05 |
|  | LVVQIDNAK |  | 51,36 | 500,297 | 998,58 | 2 | 0,004 | 0,00019 |
|  | QNQEYQVLLDVR | Gln->pyro-Glu (N-term Q) | 45,82 | 744,383 | 1486,752 | 2 | 0,01 | 7,70E-05 |
| **O77727** | 5 |  |  |  |  |  |  |  |
|  | ALEEANADLEVK |  | 94 | 651,336 | 1300,658 | 2 | 0,007 | 1,60E-07 |
|  | QSVEADINGLR |  | 92,7 | 601,316 | 1200,617 | 2 | 0,007 | 5,90E-08 |
|  | VLDELTLTK |  | 72,47 | 516,306 | 1030,598 | 2 | 0,006 | 1,80E-05 |
|  | RVLDELTLTK |  | 58,55 | 594,357 | 1186,7 | 2 | 0,008 | 0,00028 |
|  | LAADDFR |  | 49,18 | 404,204 | 806,394 | 2 | 0,002 | 0,0041 |
| **P00883** | 5 |  |  |  |  |  |  |  |
|  | GILAADESTGSIAK |  | 73,18 | 666,858 | 1331,701 | 2 | 0,008 | 6,10E-07 |
|  | ALANSLACQGK |  | 60,05 | 561,279 | 1120,543 | 2 | 0,006 | 9,90E-06 |
|  | IGEHTPSALAIMENANVLAR |  | 57,89 | 703,043 | 2106,107 | 3 | 0,017 | 2,60E-05 |
|  | VDKGVVPLAGTNGETTTQGLDGLSER |  | 57,57 | 872,128 | 2613,362 | 3 | 0,037 | 4,00E-06 |
|  | ALQASALK |  | 46,29 | 401,245 | 800,475 | 2 | 0 | 0,011 |
| **P02769** | 5 |  |  |  |  |  |  |  |
|  | KVPQVSTPTLVEVSR |  | 107,99 | 547,322 | 1638,945 | 3 | 0,015 | 4,50E-10 |
|  | LGEYGFQNALIVR |  | 56,79 | 740,407 | 1478,8 | 2 | 0,012 | 7,70E-06 |
|  | LVNELTEFAK |  | 55,13 | 582,323 | 1162,632 | 2 | 0,008 | 1,50E-05 |
|  | LVTDLTK |  | 49,37 | 395,24 | 788,465 | 2 | 0,001 | 0,00054 |
|  | QTALVELLK |  | 46,6 | 507,815 | 1013,616 | 2 | 0,004 | 0,0045 |
| **P78386** | 5 |  |  |  |  |  |  |  |
|  | LTAEIENAK |  | 60,36 | 494,77 | 987,526 | 2 | 0,003 | 7,70E-05 |
|  | LGLDIEIATYR |  | 60,07 | 632,857 | 1263,7 | 2 | 1,013 | 0,00026 |
|  | VLQAHISDTSVIVK |  | 58,95 | 503,961 | 1508,862 | 3 | 0,006 | 3,00E-06 |
|  | TKEEINELNR |  | 47,92 | 623,327 | 1244,64 | 2 | 0,004 | 0,00054 |
|  | EAECVEADSGR |  | 46,63 | 606,238 | 1210,462 | 2 | 0,003 | 0,00027 |
| **Q14525** | 5 |  |  |  |  |  |  |  |
|  | QNQEYQVLLDVR |  | 89,2 | 752,9 | 1503,785 | 2 | 0,017 | 6,50E-08 |
|  | QNHEQEVNTLR |  | 58,63 | 456,561 | 1366,662 | 3 | 0,003 | 2,40E-05 |
|  | LVVQIDNAK |  | 51,36 | 500,297 | 998,58 | 2 | 0,004 | 0,00019 |
|  | LAADDFR |  | 49,18 | 404,204 | 806,394 | 2 | 0,002 | 0,0041 |
|  | QNQEYQVLLDVR | Gln->pyro-Glu (N-term Q) | 45,82 | 744,383 | 1486,752 | 2 | 0,01 | 7,70E-05 |
| **Q9NSB2** | 5 |  |  |  |  |  |  |  |
|  | LGLDIEIATYR |  | 60,07 | 632,857 | 1263,7 | 2 | 1,013 | 0,00026 |
|  | FASFIDKVR |  | 54,47 | 541,806 | 1081,597 | 2 | 0,005 | 8,90E-05 |
|  | FASFIDKVR |  | 54,05 | 361,539 | 1081,595 | 3 | 0,003 | 0,00016 |
|  | VAPATGDLLSTGTR |  | 51,11 | 679,871 | 1357,728 | 2 | 0,008 | 5,10E-05 |
|  | FASFIDK |  | 46,78 | 414,22 | 826,426 | 2 | 0,003 | 0,0052 |
| **CRK15627.1** | 4 |  |  |  |  |  |  |  |
|  | EHALLAYTLGVK |  | 75,72 | 657,881 | 1313,748 | 2 | 0,013 | 1,20E-07 |
|  | IGGIGTVPVGR |  | 65,35 | 513,312 | 1024,609 | 2 | 0,006 | 6,90E-06 |
|  | QTVAVGVIK |  | 62,03 | 457,789 | 913,563 | 2 | 0,003 | 0,00015 |
|  | LPLQDVYK |  | 48,42 | 488,282 | 974,55 | 2 | 0,006 | 0,00094 |
| **EGY19081.1** | 4 |  |  |  |  |  |  |  |
|  | AMSILNSFVNDIFER |  | 116,09 | 878,454 | 1754,894 | 2 | 0,028 | 7,20E-11 |
|  | KETYSSYIYK |  | 72,93 | 641,323 | 1280,632 | 2 | 0,003 | 3,10E-06 |
|  | ETYSSYIYK |  | 63,83 | 577,277 | 1152,539 | 2 | 0,005 | 1,00E-06 |
|  | LILPGELAK |  | 47,62 | 477,308 | 952,602 | 2 | 0,006 | 0,0011 |
| **CRK10942.1** | 3 |  |  |  |  |  |  |  |
|  | ITDQIAQLK |  | 71,16 | 515,302 | 1028,589 | 2 | 0,002 | 2,40E-05 |
|  | NIEASVQPSR |  | 49,87 | 550,792 | 1099,569 | 2 | 0,007 | 0,0029 |
|  | IVVLEQELVR |  | 48,01 | 599,368 | 1196,722 | 2 | 0,009 | 0,0007 |
| **CRK20665.1** | 3 |  |  |  |  |  |  |  |
|  | IINEPTAAAIAYGLDKK |  | 104,06 | 596,672 | 1786,995 | 3 | 0,012 | 2,50E-10 |
|  | STAGDTHLGGEDFDNR |  | 63,54 | 564,583 | 1690,726 | 3 | 0,008 | 1,10E-06 |
|  | IINEPTAAAIAYGLDK |  | 56,72 | 830,46 | 1658,906 | 2 | 0,018 | 5,80E-06 |
| **CRK35690.1** | 3 |  |  |  |  |  |  |  |
|  | VAISGSGNVAQYAALK |  | 91,2 | 774,93 | 1547,846 | 2 | 0,015 | 1,30E-08 |
|  | VQFNSALGPYK |  | 74,73 | 612,328 | 1222,642 | 2 | 0,007 | 3,70E-07 |
|  | FLGFEQIFK |  | 59,43 | 564,812 | 1127,61 | 2 | 0,009 | 0,00042 |
| **EGY15746.1** | 3 |  |  |  |  |  |  |  |
|  | IINEPTAAAIAYGLDKK |  | 104,06 | 596,672 | 1786,995 | 3 | 0,012 | 2,50E-10 |
|  | STAGDTHLGGEDFDNR |  | 63,54 | 564,583 | 1690,726 | 3 | 0,008 | 1,10E-06 |
|  | IINEPTAAAIAYGLDK |  | 56,72 | 830,46 | 1658,906 | 2 | 0,018 | 5,80E-06 |
| **EGY19056.1** | 3 |  |  |  |  |  |  |  |
|  | GDYAIDVGR |  | 62,94 | 483,24 | 964,465 | 2 | 0,004 | 2,90E-05 |
|  | GLVGPIISR |  | 49,12 | 456,289 | 910,563 | 2 | 0,003 | 0,0012 |
|  | NVCHGSDSVENAKK |  | 47,27 | 511,899 | 1532,675 | 3 | 0,004 | 3,70E-05 |
| **O76013** | 3 |  |  |  |  |  |  |  |
|  | QNQEYQVLLDVK |  | 77,53 | 738,895 | 1475,775 | 2 | 0,013 | 1,50E-07 |
|  | LVLQIDNAK |  | 58,57 | 507,306 | 1012,597 | 2 | 0,005 | 0,00033 |
|  | LAADDFR |  | 49,18 | 404,204 | 806,394 | 2 | 0,002 | 0,0041 |
| **P00761** | 3 |  |  |  |  |  |  |  |
|  | VATVSLPR |  | 69,44 | 421,763 | 841,511 | 2 | 0,009 | 2,70E-05 |
|  | LSSPATLNSR |  | 65,49 | 523,287 | 1044,56 | 2 | 0,003 | 0,00014 |
|  | VCNYVNWIQQTIAAN |  | 50,33 | 892,428 | 1782,842 | 2 | 1,019 | 1,90E-05 |
| **P02539** | 3 |  |  |  |  |  |  |  |
|  | LSSELNSLQEVLEGYK |  | 115,12 | 904,978 | 1807,942 | 2 | 0,022 | 3,40E-11 |
|  | ATAENEFVALKK |  | 94,36 | 440,912 | 1319,713 | 3 | 0,004 | 2,30E-08 |
|  | EAECAEADSGR |  | 54,75 | 592,223 | 1182,432 | 2 | 0,004 | 7,30E-06 |
| **Q14532** | 3 |  |  |  |  |  |  |  |
|  | QNQEYQVLLDVR |  | 89,2 | 752,9 | 1503,785 | 2 | 0,017 | 6,50E-08 |
|  | LAADDFR |  | 49,18 | 404,204 | 806,394 | 2 | 0,002 | 0,0041 |
|  | QNQEYQVLLDVR | Gln->pyro-Glu (N-term Q) | 45,82 | 744,383 | 1486,752 | 2 | 0,01 | 7,70E-05 |
| **Q9NSB4** | 3 |  |  |  |  |  |  |  |
|  | LAGLEEALQK |  | 71,91 | 536,811 | 1071,608 | 2 | 1,011 | 2,70E-05 |
|  | LGLDIEIATYR |  | 60,07 | 632,857 | 1263,7 | 2 | 1,013 | 0,00026 |
|  | AQYDDIASR |  | 46,72 | 519,749 | 1037,482 | 2 | 0,005 | 0,00053 |
| **CRK05543.1** | 2 |  |  |  |  |  |  |  |
|  | FADLSEHNYGVSILNDSK |  | 78,83 | 670,332 | 2007,975 | 3 | 0,021 | 4,40E-08 |
|  | RPTHYNTSWDMAK |  | 47,84 | 536,255 | 1605,743 | 3 | 0,007 | 3,30E-05 |
| **CRK11434.1** | 2 |  |  |  |  |  |  |  |
|  | YIGELELNR |  | 48,9 | 553,799 | 1105,584 | 2 | 0,007 | 0,00051 |
|  | FSTVLGSR |  | 46,43 | 433,742 | 865,469 | 2 | 0,003 | 0,0012 |
| **CRK35694.1** | 2 |  |  |  |  |  |  |  |
|  | VAISGSGNVAQYAALK |  | 91,2 | 774,93 | 1547,846 | 2 | 0,015 | 1,30E-08 |
|  | FLGFEQIFK |  | 59,43 | 564,812 | 1127,61 | 2 | 0,009 | 0,00042 |
| **EEY15502.1** | 2 |  |  |  |  |  |  |  |
|  | LLLIGDSGVGK |  | 57,48 | 536,328 | 1070,641 | 2 | 0,008 | 0,00013 |
|  | LQIWDTAGQER |  | 52,53 | 658,839 | 1315,664 | 2 | 0,012 | 5,20E-05 |
| **EEY16611.1** | 2 |  |  |  |  |  |  |  |
|  | VVDASVYPR |  | 66,78 | 503,274 | 1004,533 | 2 | 0,004 | 2,60E-06 |
|  | ATGVEFLDGQYLYK |  | 50,67 | 802,411 | 1602,807 | 2 | 0,014 | 7,40E-05 |
| **EEY17079.1** | 2 |  |  |  |  |  |  |  |
|  | VLPAPSSLWLDATGK |  | 87,13 | 777,934 | 1553,853 | 2 | 0,008 | 6,00E-08 |
|  | VPEHFVVGVPAHVDGR |  | 45,89 | 429,483 | 1713,901 | 4 | 0,006 | 5,00E-05 |
| **EEY17295.1** | 2 |  |  |  |  |  |  |  |
|  | FTQAGSEVSALLGR |  | 74,38 | 718,388 | 1434,761 | 2 | 0,014 | 6,40E-07 |
|  | VVDLLAPYAR |  | 47,34 | 558,827 | 1115,64 | 2 | 0,006 | 0,00014 |
| **EEY17447.1** | 2 |  |  |  |  |  |  |  |
|  | IVLGMPLYGR | Oxidation (M) | 61,27 | 567,825 | 1133,635 | 2 | 0,009 | 2,10E-05 |
|  | IVLGMPLYGR |  | 48,38 | 559,827 | 1117,64 | 2 | 0,008 | 0,00053 |
| **EGY14777.1** | 2 |  |  |  |  |  |  |  |
|  | LIAGGAAQNSAR |  | 71,76 | 564,812 | 1127,609 | 2 | 0,005 | 2,40E-05 |
|  | LSIQELGPSYPFPK |  | 59,11 | 788,93 | 1575,846 | 2 | 1,011 | 2,90E-06 |
| **EGY15999.1** | 2 |  |  |  |  |  |  |  |
|  | LLLIGDSGVGK |  | 57,48 | 536,328 | 1070,641 | 2 | 0,008 | 0,00013 |
|  | LQIWDTAGQER |  | 52,53 | 658,839 | 1315,664 | 2 | 0,012 | 5,20E-05 |
| **EGY17622.1** | 2 |  |  |  |  |  |  |  |
|  | ALGDLLELDDTVR |  | 92,09 | 715,387 | 1428,759 | 2 | 0,013 | 4,90E-08 |
|  | QMHALDYDR | Gln->pyro-Glu (N-term Q) | 47,16 | 566,251 | 1130,487 | 2 | 0,005 | 0,0017 |
| **O76011** | 2 |  |  |  |  |  |  |  |
|  | QNQEYQVLLDVR |  | 89,2 | 752,9 | 1503,785 | 2 | 0,017 | 6,50E-08 |
|  | QNQEYQVLLDVR | Gln->pyro-Glu (N-term Q) | 45,82 | 744,383 | 1486,752 | 2 | 0,01 | 7,70E-05 |
| **O76014** | 2 |  |  |  |  |  |  |  |
|  | QNQEYQVLLDVK |  | 77,53 | 738,895 | 1475,775 | 2 | 0,013 | 1,50E-07 |
|  | LAADDFR |  | 49,18 | 404,204 | 806,394 | 2 | 0,002 | 0,0041 |
| **O76015** | 2 |  |  |  |  |  |  |  |
|  | QNQEYQVLLDVK |  | 77,53 | 738,895 | 1475,775 | 2 | 0,013 | 1,50E-07 |
|  | LAADDFR |  | 49,18 | 404,204 | 806,394 | 2 | 0,002 | 0,0041 |
